# A Control Method with Reinforcement Learning for Urban Un-Signalized Intersection in Hybrid Traffic Environment

**DOI:** 10.3390/s22030779

**Published:** 2022-01-20

**Authors:** Yanjun Shi, Yuanzhuo Liu, Yuhan Qi, Qiaomei Han

**Affiliations:** 1 School of Mechanical Engineering, Dalian University of Technology, Dalian 116024, China; yuanzhuo.liu@foxmail.com (Y.L.); qiyuhan.dut@foxmail.com (Y.Q.); 2 Department of Electrical and Computer Engineering, Western University, London, ON N6A 5B9, Canada; qhan42@uwo.ca

**Keywords:** reinforcement learning, connected and autonomous vehicles, urban unsignalized intersection

## Abstract

To control autonomous vehicles (AVs) in urban unsignalized intersections is a challenging problem, especially in a hybrid traffic environment where self-driving vehicles coexist with human driving vehicles. In this study, a coordinated control method with proximal policy optimization (PPO) in Vehicle-Road-Cloud Integration System (VRCIS) is proposed, where this control problem is formulated as a reinforcement learning (RL) problem. In this system, vehicles and everything (V2X) was used to keep communication between vehicles, and vehicle wireless technology can detect vehicles that use vehicles and infrastructure (V2I) wireless communication, thereby achieving a cost-efficient method. Then, the connected and autonomous vehicle (CAV) defined in the VRCIS learned a policy to adapt to human driving vehicles (HDVs) across the intersection safely by reinforcement learning (RL). We have developed a valid, scalable RL framework, which can communicate topologies that may be dynamic traffic. Then, state, action and reward of RL are designed according to urban unsignalized intersection problem. Finally, how to deploy within the RL framework was described, and several experiments with this framework were undertaken to verify the effectiveness of the proposed method.

## 1. Introduction

**Figure 1:**
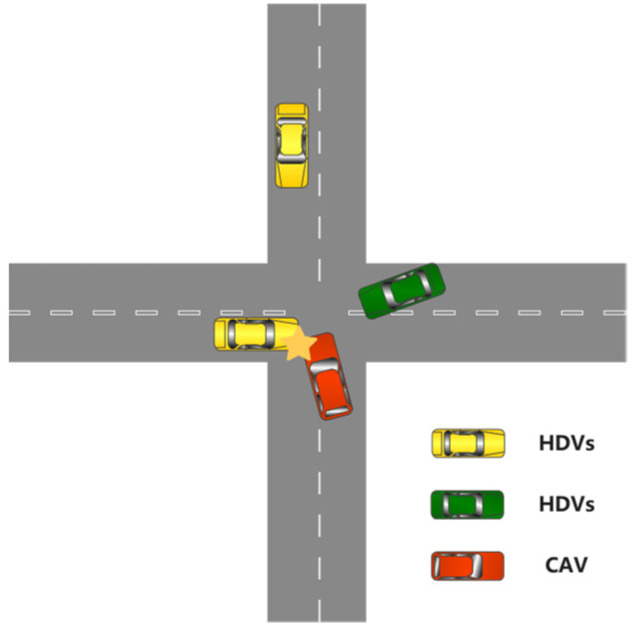
Connected and automated vehicle (CAV) (red) and human driving vehicles (HDVs) (yellow and green) will collide when they cross an unsignalized intersect.

The world city population has increased to 55% in the past decades, and is expected to increase to 68% in 2050, leading to an intensification of traffic congestion affecting safety [1]. In addition, according to the investigation, 6 million traffic accidents occurred in the United States in 2018, causing more than 3.5 million deaths and 2.5 million injuries. Therefore, vehicle coordination and management are worth studying to improve the security and efficiency of future transportation systems in complex conditions, especially in urban environments. Today, there are many efforts to solve the problems, such as (a) research about the traffic structure, and (b) research about the autonomous vehicle. Difficulties in automatic driving at crossroads are mainly due to complex traffic conditions. The no-signal intersections are more complicated and challenging for the city view, involving multi-vehicle interaction [2,3,4]. Therefore, this paper specifically discusses autonomous driving in the unsignalized intersection, while considering a basic scenario where human drivers and autonomous vehicles coexist, as shown in Figure 1. 

Autonomous navigation technology has two types: sensor and communication type. In sensor systems using a camera, radar and laser radar can directly detect the surrounding obstacles. However, that has a limited smaller detection area when the obstacle hinders the sensor. The limitations of sensors can be broken by vehicles and everything (V2X) communication technology. V2X refers to a technique by which the vehicle exchanges information with other vehicles, roads, and other infrastructure by a wireless network [1]. V2X, as a new technology, not merely provides a safer and more comfortable traffic environment, but is important for reducing accident rates, reducing pollution, and improving traffic efficiency. With the development of V2X communication technology, information sharing and vehicle coordination between connected and automated vehicles (CAVs) and connected vehicles (CVs) improves safety and efficiency [5]. As aforementioned, this provides the possibility for the safety and efficiency of no-signal intersections. However, in an autonomous vehicles problems in urban traffic environments with unsignalized intersections, rule-based, optimization-based, learning-based and so on were proposed. 

Traditional intersections (such as signal intersections) are not necessarily the best strategy in the CAVs environment. Rule-based methods have been proposed by Zhang [6] and Zhao [7] which have been used to solve different traffic scenarios based on the first-in-first-out (FIFO) rule. Dresner and Stone [8] introduced a reservation-based scheme that requires CAVs to reserve a space-time slot inside the intersection. Lee and Park [9] minimized the total length of overlapped trajectories of CAVs crossing an intersection. Gregoire et al. [10] decomposed the coordination problem into a central priority assignment and trajectory planning. It has been proven that FCFS-based A.I.M. and its variants can reduce delay and emissions compared to traditional signal control under certain traffic conditions (Fajardo et al., 2011; Li et al., 2013) [11].

The vehicle interaction is modelled as a dynamic system, from the action of the control vehicle as an input in the optimal control setting. An online model predictive control (MPC) method is presented by Borek et al. [12], this model is mainly optimal for the energy of heavy trucks, which uses the best solution to track using dynamic program offline. Du et al. [13] presented a three-layered hierarchical coordination strategy for CAVs at multiple intersections. Although experiments have proven well, the MPC-based approach relies on precise dynamic merge models (including human driving models), which typically require calculations because each step requires online optimization [14].

However, data driving methods such as reinforcement learning (RL) are increasingly concerned, and they have been explored in automatic driving roads. The integration of Deep Learning (DL) and RL, widely referred to as Deep Reinforcement Learning (DRL), has shown its potential by successfully solving video games [15], 3D locomotion [16], Go games [17] and many other problems. Vinitsky et al. [18] proposed a merging strategy via reinforcement learning to control shockwaves from on-ramp merges, which is similar to the unsignalized intersections scenario. In the literature, there are relatively few studies using RL to solve AIM problems. Isele et al. [19] proposed a single-agent RL approach to navigate one autonomous vehicle through the intersection.

In general, automatic driving vehicles are autonomous agents that use advanced communication technologies and sensors to perceive and interact with real-time traffic conditions (environment). Our research framework, RL, has been proposed to solve Markov decision processes (MDPs). Generally, in RL, agents learn the optimal policy by trial-and-error interaction with the dynamic environment formally described by MDPs. RL combined with deep learning has achieved outstanding success in various areas such as video games [20] and robotics [21]. These advances have inspired the research community to examine the performance of deep reinforcement learning in autonomous driving [22]. So, in this paper, we explore the RL’s ability to combine radars, LiDAR, cameras, sensors, and V2X for autonomous driving at unsignalized intersections.

While the rule-based method works well in simple scenarios, it can be very unstable in complex environments [23]. An optimal strategy is often related to computational complexity issues because online optimization is required at each time step [14]. However, for dynamic systems such as Autonomous intersection management (AIM), the traffic environment changes over time, and predefined strategies may become unsatisfactory, especially when there is a great deal of uncertainty in a hybrid traffic environment. In this paper, our goal is to propose an optimal framework that fully considers the real-time traffic dynamics of the vehicle.

Little attention has been paid to a truly autonomous vehicle in a complex urban environment. Most of the previous studies are based on macro-control, and few are based on micro discussion. On the other hand, many studies aim at either all connected and automated or multiple vehicles. There is very little discussion of only one connected and autonomous vehicle (CAV) and many human driving vehicles (HDVs). To fill these gaps, we proposed a proximal policy optimization (PPO) advanced algorithm-based V2X. The main contributions of this paper are as follows:

- We propose an intelligent transportation system for the Internet of vehicles based on 5G, edge and cloud computing technologies. Moreover, the proposed framework solves the fine-grained problem of automatic driving vehicles in hybrid traffic.
- We describe an RL problem in the urban unsignalized intersection of traffic issues (a CAV and HDVs coexist on the intersection). In our paper, we consider a dynamic environment that has a time-varying connectivity topology.
- This paper proposes deployment algorithms, increasing the possibility of automatic driving vehicles in a real traffic environment.
- In the experimental part, we used a variety of different performance measurement methods to measure the algorithm’s performance and ablation experiments.

## 2. Research Methodology

### 2.1. VRCIS (Vehicle-Road-Cloud Integration System)

The Vehicle-Road-Cloud Integration System (VRCIS) uses a new generation of information and communication technology to connect the physical, information, and application layers of people, vehicles, roads, and clouds. As a whole, a cyber-physical system that integrates perception, decision-making, and control can realize the comprehensive improvement of vehicle driving and traffic operation safety and efficiency. It can also be called “Intelligent Networked Vehicle Cloud Control System”, or simply “Cloud Control system”.

**Figure 2:**
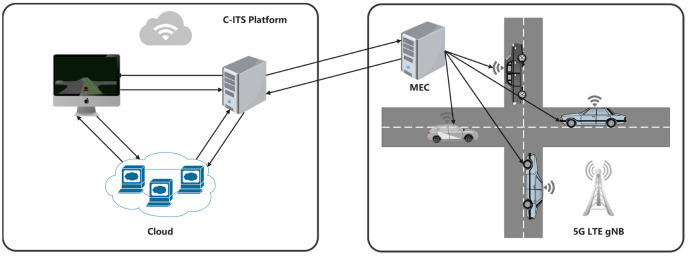
Vehicle-Road-Cloud Integration System (VRCIS). The left part of the figure is the cloud platform where the algorithm is trained, and the right part is a real traffic scenario where the algorithm is developed. The system communicates through vehicles and everything (V2X) technology.

Figure 2 shows an architecture of cloud edge collaboration in the unsignalized intersection scenario, which consists of the Cloud Server, Edge devices (include RoadSide Unit (RSU) and OnBoard Unit (OBU)) and Vehicles Interactors. CAV and other vehicles information (speed, location, and so on) can be observed and shared using V2X technology, and both cloud and edge servers are equipped with powerful GPU resources for neural network training. In this proposed scheme, we use Dedicated Short Range Communications (DSRC) and Long Term Evolution (LTE-V) technology, making sure to communicate between CAV with HDVs, and vehicles and Mobile Edge Computing (MEC) communicated with Cloud.

### 2.2. Longitudinal Dynamic Models

The intelligent driver model (I.D.M.), as a stand car-following model, is used in our work, describing the dynamics of single vehicles’ positions and velocities. The I.D.M. is a time-continuous car-following model for highway and urban traffic simulation. Treiber, Hennecke and Helbing developed it in 2000 to improve the results of other “intelligent” drive models, such as Gipps’ model, the latter lost realistic attributes under certainty limits [24].

(1) $$v_{i}=\dot{x}=\frac{dx_{i}}{dt}$$

(2) $$a_{IDM}={\dot{v}}_{i}=\frac{dv_{i}}{dt}=a\left(1-{\left(\frac{v_{i}}{v_{0}}\right)}^{\delta }-{\left(\frac{s^{*}\left(v_{i},\Delta v_{i}\right)}{s_{i}}\right)}^{2}\right)$$

(3) $$s^{*}\left(v_{i},\Delta v_{i}\right)=s_{0}+v_{i}T+\frac{v_{i}\Delta v_{i}}{2\sqrt{ab}}$$

(4) $$s_{i}:=x_{i-1}-x_{i}-l_{i-1}$$

(5) $$\Delta v_{i}:=v_{i}-v_{i-1}$$

These parameters are represented in Table 1. Table 2 shows the required parameters in the intelligent driving model.

### 2.3. Proximal Policy Optimization

#### 2.3.1. Background

In this section, we reviewed basic theory about RL to understand the model proposed.

Reinforcement Learning (RL) is a subsequent field of machine learning. It is concerned about how agents interact with the environment and learn to maximize the accumulation return. In RL problems, it is often regarded as an infinite-horizon discounted MDP, defined by the quintuple (S,A,P,R,γ), where s∈S is a set of states and s is a specific state; where a∈A is a set of actions and a is a specific action; $P(S,\;a,\;S^{\prime })$ defines a probability for a transition from S to $S^{\prime }$ by an action; R(s,a) defines the immediate reward for taking action.; γ∈[0,1] defines the discount factor. In order to maximize some cumulative rewards function, we try to seek to learn optimal policy $\pi^{*}$, where the policy is a stochastic policy π: S×A→[0,1], typically the expected discounted sum over a potentially infinite horizon from each state following policy π:

(6) $$v^{\pi }(s)=E_{a_{t},s_{t+1},\ldots }\left\{\sum_{l=t}^{\infty }\gamma^{l-t}r_{l}|s_{t}=s\right\}$$

where $a_{t}∼\pi \left(a_{t}|s_{t}\right),s_{t+1}∼p\left(s_{t+1}|s_{t},a_{t}\right)$ and $r_{t}∼r\left(s_{t},a_{t},s_{t+1}\right)$. Alternatively, the definition of a state function $q^{\pi }(s,a)$ is expressed as follows:

(7) $$q^{\pi }(s,a)=E_{s_{t+1},a_{t+1},\ldots }\left\{\sum_{l=t}^{\infty }\gamma^{l-t}r_{l}|s_{t}=s,a_{t}=a\right\}$$

However, in the proposed problem, a CAV interacts with the environment, which is just possible to model an MDP model. Our objective is to maximize a reward function to an autonomous vehicle walking as human driving in an unsignalized intersection by improving a policy.

#### 2.3.2. Proximal Policy Optimization Advanced

Since trust region policy optimization (TRPO) [25] is relatively complicated and we still want to implement a similar constraint, PPO simplifies it by using a clipped surrogate objective while retaining similar performance.

In this work, model-free reinforcement learning methods are used to optimize the control policy in the unsignalized intersection. For policy-based RL algorithms, we compute an estimator for the policy gradient as follows:

(8) $$\nabla \theta =\hat{E}\left[\nabla_{\theta }\log\pi_{\theta }\left(a_{t}\;|\;s_{t}\right)A_{t}\right]$$

Here, action *a* per time step *t* is controlled the parameterized policy $\pi_{\theta }\left(a_{t}\;|\;s_{t}\right)$ under the state *s*, and update the parameter θ to maximize the cumulative reward. Where $\hat{E}\left[\ldots \right]$ denotes the empirical average over a finite batch of samples and $A_{t}$ denotes the advantage function. The loss function for updating a RL policy to estimate the policy gradient has the form as:

(9) $$L^{PG}(\theta )=\;{\hat{E}}_{t}\left[\log\pi_{\theta }\left(a_{t}|s_{t}\right){\hat{A}}_{t}\right]$$

Firstly, the probability ratio is denoted between old and new policies as:

(10) $$r(\theta )=\frac{\pi_{\theta }(a\;|\;s)}{\pi_{\theta_{old}{}_{\;}}(a\;|\;s)}$$

Then, the objective function of TRPO becomes:

(11) $$J^{\mathrm{TRPO}}(\theta )=E\left[r(\theta ){\hat{A}}_{\theta_{\mathrm{old}}}(s,a)\right]$$

Without a limitation on the distance between $\theta_{old}$ and $\theta_{}$, maximizing $J^{\mathrm{TRPO}}(\theta )$ would lead to instability with extremely large parameter updates and big policy ratios. PPO imposes the constraint by forcing r(θ) to stay within a small interval around 1, precisely [1−ϵ,1+ϵ], where ϵ is a hyperparameter.

(12) $$J^{\mathrm{CLIP}}(\theta )=E\left[\min\left(r(\theta ){\hat{A}}_{\theta_{\mathrm{old}}}(s,a),clip(r(\theta ),1-\epsilon ,1+\epsilon ){\hat{A}}_{\theta_{\mathrm{old}}}(s,a)\right)\right]$$

Network architecture applying PPO with shared parameters for policy (actor) and value (critic) functions. In addition to the clipped reward, the objective function is augmented with an error term on the value estimation $V_{\theta }(s)$ and an entropy term $H\left(s,\pi_{\theta }\right)$ to encourage sufficient exploration.

(13) $$J^{{\mathrm{CLIP}}^{\prime }}(\theta )=E\left[J^{\mathrm{CLIP}}(\theta )-c_{1}{\left(V_{\theta }(s)-V_{\mathrm{target}}\right)}^{2}+c_{2}H\left(s,\pi_{\theta }\right)\right]$$

where both $c_{1}$ and $c_{2}$ are two hyperparameter constants, based on the above formula, the flow chart of our proposed algorithm is shown in Algorithm 1.

The advantage function ${\hat{A}}_{\theta_{\mathrm{old}}}(s,a)$ can be defined as a way of measuring how much we can improve by taking action in a particular state. We want to use the reward at each time step and calculate how much advantage can be gained by taking action, not only in the short term but also by focusing on a longer time. In order to calculate this, Generalized Advantage Estimation (GAE) [26] is used.

(14) $${\hat{A}}_{t}^{}=\sum_{l=1}^{\infty }{\left(\gamma \lambda \right)}^{l}\delta_{t+l}^{V}=\sum_{l=1}^{\infty }{\left(\gamma \lambda \right)}^{l}\left(r_{t}+\gamma V\left(s_{t+l+1}\right)-V\left(s_{t+l}\right)\right)$$

where γ hyperparameter content is called a discount factor to reduce the value of the future state, since we want to emphasize more on the current state than a future state; where lambda is a smoothing hyperparameter content used for reducing the variance in training which makes it more stable. The parameter λ is suggested for 0.99 and the parameter lambda is suggested for 0.95.
**Algorithm 1** PPO with Clipped Objective.1: Input: initialize policy parameters $\theta_{0}$, initialize ϵ
2: For *k* = 0,1,2, … do3: Use policy $\pi_{\theta }$, to collect trajectories $D_{k}=\left\{\tau_{i}\right\}$ by the environment4: Compute ${\hat{R}}_{t}$ (rewards-to-go)5: Compute advantage estimates ${\hat{A}}_{t}$ using GAE (advantage estimation algorithm)6: Update the policy:7:       $\theta_{k+1}=\arg\max_{\theta }J_{\theta^{k}}^{\mathrm{CLIP}}(\theta )$
8: Take K steps of minibatch SGD (Adam), where:9:  $J_{\theta_{k}}^{CLIP}(\theta )=\underset{\tau ∼\pi_{k}}{E}\left[\sum_{t=0}^{T}\left[\min\left(r_{t}(\theta ){\hat{A}}_{t}^{\pi_{k}},clip\left(r_{t}(\theta ),1-\epsilon ,1+\epsilon \right){\hat{A}}_{t}^{}\right)\right]\right]$
10: End for

### 2.4. RL Formulation

**Figure 3:**
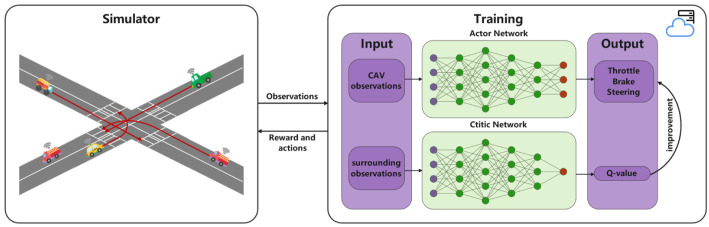
The neural network structure of PPO.

In this section, we intend to transform an unsignalized intersection problem into an RL problem. First, the simulation scene is modelled as the Markov model. Secondly, the proposed algorithm controls the CAV to complete the automatic driving. In Figure 3, the observations returned by the simulator serve as the input of the algorithm proposed in this paper. Then, the algorithm outputs the optimal continuous actions (throttle, brake and steering) to control the behavior of CAV, and finally safely arrives at the specified location. So, defining state, action and reward is of such importance. 

#### 2.4.1. State Space

In this study, a state-space represents information about the CAV and surrounding social vehicles, such as the vehicle’s steering, heading, speed and position, obtained by V2X technology. We define the state space in Table 3.

#### 2.4.2. Action Space

Unlike most studies on autonomous driving, we designed the motion space to be continuous. For example, if the CAV has a vehicle in front of it, does it accelerate to pass it by throttling and steering, or does it brake? It is all guided by the algorithms that we have provided. As shown in Table 4, the action space is defined as follows:

#### 2.4.3. Reward Function

As the most critical factor, the reward function for optimal convergence policies in RL The purpose of the reward is to maximize discount returns. Our purpose is to avoid collisions safely, comfortably and quickly the goal. The specific part of each part of the reward function is designed as follows.

(1) Safety: To avoid collisions. In terms of safety, the penalty function of the CAV for collisions can be expressed as:

(15) $$r_{collision}(s_{t},a_{t})=\{\begin{array}{c} -c,\\ 0, \end{array}\begin{array}{c} if\;True\\ else\;False \end{array}$$

where *c* is the absolute value of the penalty factor, this function tells us that we should minimize the number of collisions in the end.

(2) Comfort: Smaller jerk (angular jerk and linear jerk). In respect of comfort, the penalty function of the CAV for comfort can be represented

(16) $$r_{\mathrm{angular}\_\mathrm{jerk}}=-c_{1}*a_{\mathrm{angular}\_\mathrm{jerk}}$$

(17) $$r_{\mathrm{linear}\_\mathrm{jerk}}=-c_{2}*a_{\mathrm{linear}\_\mathrm{jerk}}$$

where *c*_1_ and *c*_2_ are the absolute value of the penalty factor; $a_{\mathrm{angular}\_\mathrm{jerk}}$ and $a_{\mathrm{linear}\_\mathrm{jerk}}$ are the lateral jerk and the longitudinal jerk. To avoid sudden acceleration or deceleration of vehicles, the vehicle occupant may not be discomfort in this reward function.

(3) Efficiency: Get to the target quickly. In terms of safety, the penalty function of the CAV for efficiency can be expressed as

(18) $$r_{\mathrm{reached}\_\mathrm{goal}}(s_{t},a_{t})=\{\begin{array}{c} c_{3},\\ 0, \end{array}\begin{array}{c} \mathrm{if}\;\mathrm{True}\\ \mathrm{else}\;\mathrm{False} \end{array}$$

(19) $$r_{\mathrm{speed}\_\mathrm{reward}}(s_{t},a_{t})=\min(0,c_{4}(v_{\max\_\mathrm{speed}}-v_{\mathrm{current}}))$$

where *c*_3_ is the absolute value of the penalty factor; $v_{\mathrm{current}}$ and $v_{\max\_\mathrm{speed}}$ are current speed and the max limit speed. We hope that CAV can reach the goal smoothly and quickly every time, so when it reaches the goal, give a larger reward.

Thus, the complete form of reward function is

(20) $$r_{\mathrm{total}}=r_{\mathrm{collision}}+r_{\mathrm{reached}\_\mathrm{goal}}+r_{\mathrm{off}\_\mathrm{road}}+r_{\mathrm{speed}\_\mathrm{reward}}$$

(4) Termination

The Termination condition of an episode in reinforcement learning, when ‘Termination = True’ means that the environment needs to be reset, CAV will be randomly generated at a point again to continue the training of the loop.

When the collision is True, the agent ends an episode and then continues the next loop training.

### 2.5. Framework and Development

**Figure 4:**
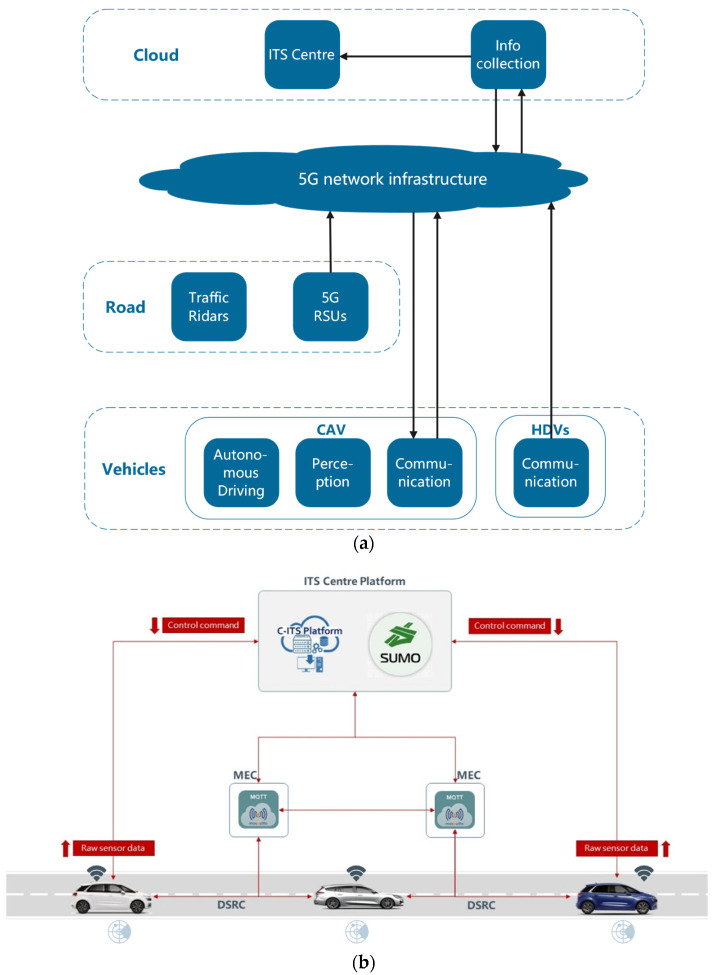
Shows how the training and deployment phases are implemented; (**a**) Training architecture. (**b**) Development architecture.

In the section, a scheme is described at the system level and it is shown in Figure 4. The implementation of the framework includes two phases: the training phase and the deployment phase. The CAV is first trained with T intersection and Cross intersection in SMARTS, then, which is ported to the Cross intersection, connected to the real scenario with RSU, it starts to control the CAV.

(1) Training phase

The CAV is trained by interacting with the simulator. The simulator randomly generates social vehicles to arrive and specifies a CAV to drive from south to west. It obeys traffic rules and interacts with surrounding social vehicles. The simulator obtains the state, calculates the current reward rt accordingly, and provides it to the CAV. Using the Policy-Gradient update formula referenced in the previous sections, the agent updates itself based on the information from the simulator. At the same time, Ent selects an at action (throttle, steering, brake) and forwards the action to the simulator. Then the simulator will update and change the physical state of the CAV. The steps are repeated until convergence, and the agent is trained. 

The agent’s performance is largely dependent on the quality of the simulator. In order to be similar to the real world, the emulator is randomly generated according to the real crossroads. To solve the difference in traffic flow at different times in a day, we granulate according to the traffic density so that the agent can adapt to different traffic flows at different times during training.

The training goal is to make the CAV smooth, safe and fast from the starting point to the finishing point, without colliding with the social vehicle or driving off the track.

As Figure 4a, the vehicle’s information is collected and transmitted to the Cloud. In addition we were training on the Cloud through the PPO algorithm. 

(2) Development

In the deployment phase, the trained agent CAV is migrated to the intersection for vehicle control and installed the software agent to the road test edge device to implement the control of the automatic driving vehicle. Here, the agent does not update the learned J(θ) but controls the CAV. V2X provides the state of the current environment, and CAV selects three consecutive actions based on the trained v-network based on state. This step is executed in real-time to achieve continuous vehicle control.

## 3. Experiment and Evaluation

**Figure 5:**
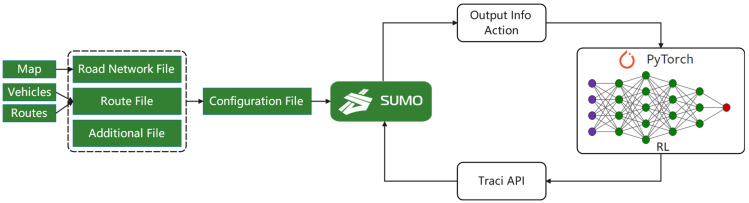
Autonomous driving simulation theory framework.

In this section, to verify the effectiveness of the proposed algorithm, we have carried out many simulation experiments and analyses. We use the SMARTS (Scalable Multi-Agent RL Training School) simulator, open-source software for autonomous driving simulation based on Pybullet and SUMO [27]. The role of Pybullet is to perform physical dynamics simulation rendering of SUMO to make it closer to the real environment in Figure 5. The Pytorch framework is used for neural network inference training to get the actions we want in this experiment. Use Python to call SUMO’s Traci API to control the entire traffic system to simulate reality.

### 3.1. Experimental Settings

The proposed algorithm is trained in the 2lane and 3lane Cross intersection, and in the same way, evaluated in the Cross intersection. We use low, medium and high traffic density levels, as is shown in Table 5. There are three different speed limits in three separate traffic densities, shown in Table 6.

Select appropriate variables for training through hyperparameter adjustment. In the proposed scheme, the PPO algorithm uses a neural network to simulate collision-free rules, minimizing the action loss between the neural network and the rules. There is only one neural network, including three dense layers and two normalization layers. In the hidden layer, ReLU is selected as the activation function. The PPO using the adaptive KL penalty algorithm controls the distance between the update strategy and the old strategy to avoid noise in the gradient update process. Therefore, PPO hyperparameter initialization improves the effectiveness of RL in various tasks. This study proposes a PPO hyperparameter set for hybrid autonomous traffic at un-signalized intersections, as shown in Table 7.

### 3.2. Performance

In this section, we show the performance of our algorithm to analyze the experimental results, and a lot of metrics are used.

**Figure 6:**
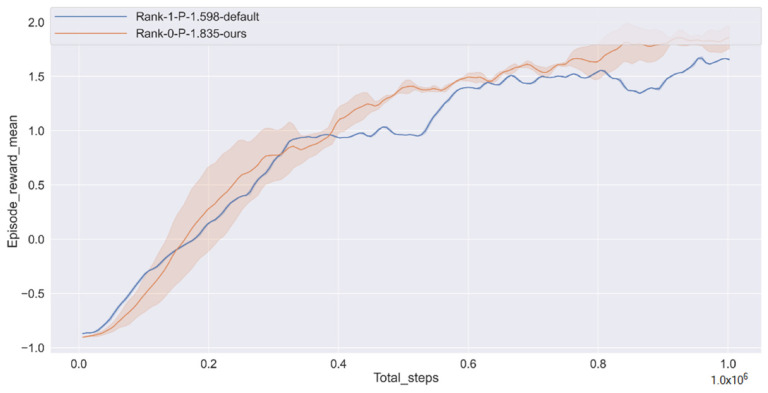
Episode cumulative mean reward.

In reinforcement learning, the curve of the reward function is the most important index to measure the algorithm. Figure 6 shows the stable convergence of our proposed algorithm. Compared with the default algorithm at SMARTS, the algorithm has increased by 14.8%.

**Figure 7:**
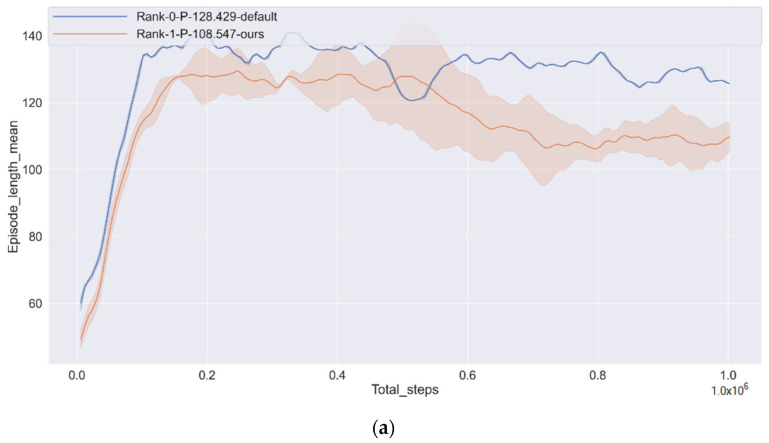

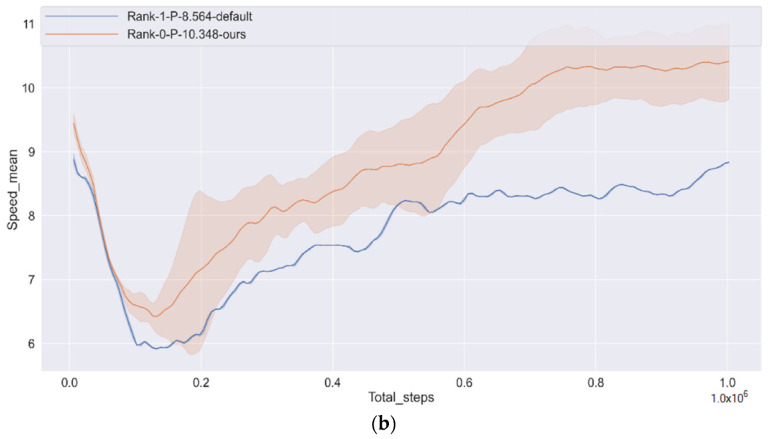
The length of the episode of each step is getting shorter and shorter, faster and faster. (**a**) Episode length mean; (**b**) Speed mean.

Figure 7a visualizes the mean episode length during the total steps, and both the default and ours are increasing rapidly. From Figure 7b, the mean speed curve is lowered first, so episode length increases in Figure 7a. The tendency can explain that the policy learns how to avoid collision carefully because the CAV gets a larger negative reward. However, both Figure 6 and Figure 7a show the decreasing tendency of the curve after 20,000 steps, which can be explained that CAV has learned how to avoid collision and reach the target quickly. Among them, the mean length of the episode is reduced by 15.48% than the default, and the mean speed is 20.83% higher than the default.

**Figure 8:**
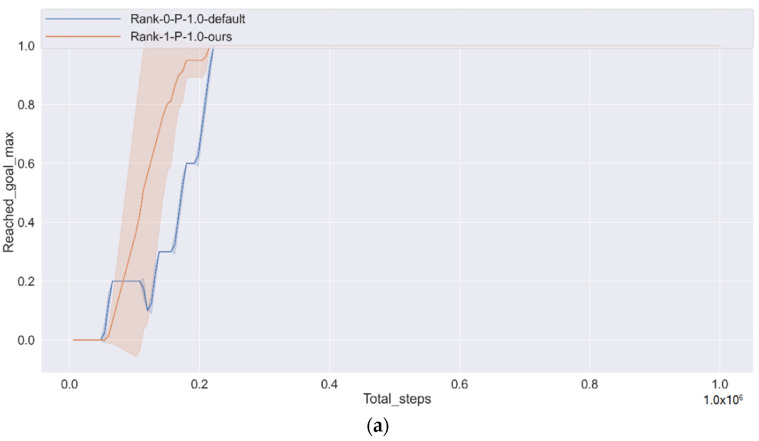

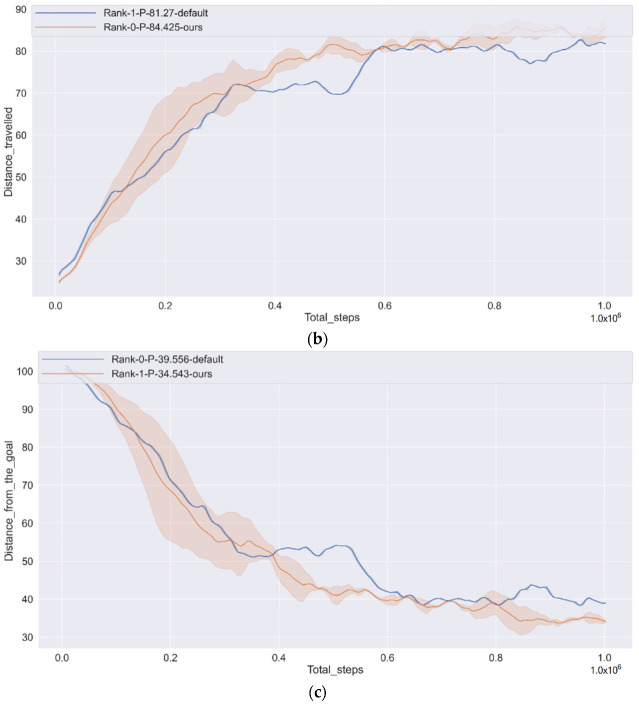
Performance of effectiveness total steps in experiment, including reach the goal max rate, distance travelled and distance from the goal. (**a**) Reached goal max rate; (**b**) Distance travelled; (**c**) Distance from the goal.

Figure 8a shows a condition of reaching a goal over time. Moreover, the performance metrics here maximize the goal’s reaching ratio rather than average because we think it is more intuitive. The algorithm proposed in this article has learned how to reach the goal. Figure 8b,c show the CAV getting closer to the goal, and its travel distance is growing, respectively. It can be observed from Figure 8b that the travel distance of our algorithm is higher than the default at almost every time step, which is 3.88% higher on average.

**Figure 9:**
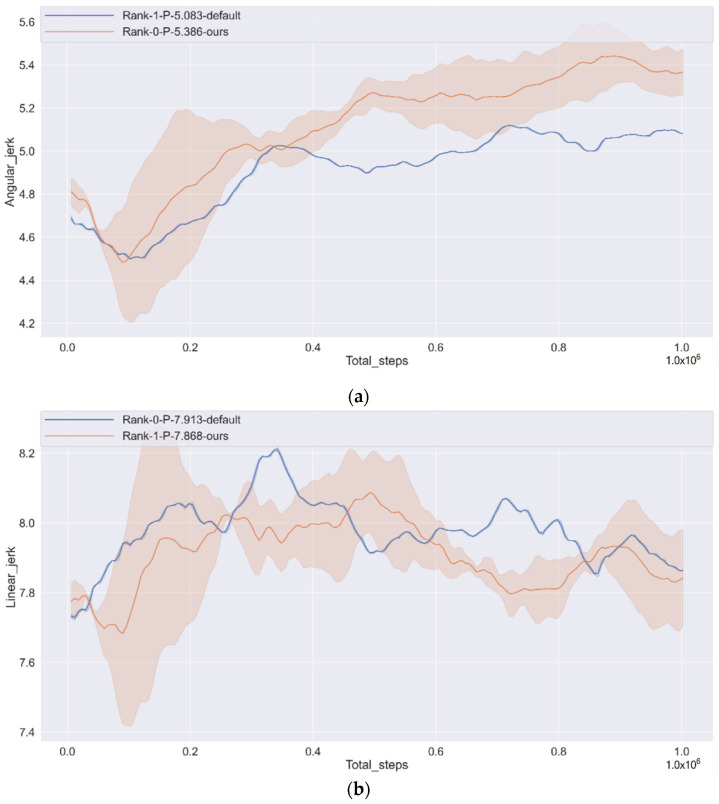
CAV changes in steering angle and linear acceleration during driving. (**a**) Angular jerk; (**b**) Linear jerk.

Figure 9a,b show the angular jerk and linear jerk changes. Based on the above figure, the angular acceleration increases by 5.96% from the default, and the linear acceleration ratio increases by 0.57% by default. We can see the angular jerk change is relatively gentle, but linear jerk changes are uniform since the CAV sacrifices comfortable performance to learn how to secure and quickly reach the goal. However, we consider this problem in the reward function and believe that the next work will solve this problem.

## 4. Conclusions

In this paper, we defined the problem of autonomous driving at an unsignalized intersection in mixed traffic as on-policy RL and developed an effective RL algorithm. In addition, we proposed an VRCIS framework based on V2X. The CAV has more perception capabilities and can make more precise decisions under this frame. For the PPO algorithm and framework above, we also gave the training algorithm and the way to deploy the algorithm to make safe autonomous driving closer to reality.

We finally noted several limitations of our work. First, we need to consider the effects between neighboring intersections to apply our protocol in the real world. Secondly, the CAV may transmit incorrect information with V2V communications. In future work, we will design more secure intersection protocols against false data provided by CAVs. In addition, we will pay attention to other traffic scenarios and plan to test the algorithm in real-life scenarios.

## Figures and Tables

**Table 1:** Parameters list.

Symbol	Meaning
$v_{i}$	The leading vehicle’s speed
$\dot{x}$	The derivative of displacement (*x*)
*v*_0_	The desire velocity
δ	The acceleration exponent
$s^{*}\left(v_{i},\Delta v_{i}\right)$	The desired headway
$s_{i}$	The headway between vehicles
$\Delta v_{i}$	The difference between the velocity and the lead velocity
*s*_0_	The minimum spacing
*T*	The desire time headway
$a_{IDM}$	Acceleration
*a*	The acceleration term
*b*	The comfortable braking deceleration

**Table 2:** Typical parameters of an intelligent driver model (I.D.M.) in the context of city traffic.

Parameters	Value
Desired Speed (m/s)	15
Time gap (s)	1.0
Minimum gap (m)	2.0
Acceleration exponent	4.0
Acceleration (m/s^2^)	1.0
Comfortable acceleration (m/s^2^)	1.5

**Table 3:** The composition of the state space.

Name	Explain
Speed	The current speed of a CAV.
Steering	The current steering of a CAV.
Heading	Heading angle of a lane at this point (radians)
Position	The current position of a CAV.
Collisions	Whether there is a collision between CAV and other vehicles
Off the Road	Whether the CAV vehicle is off the road

**Table 4:** The composition of the action space.

Name	Scope
Throttle	[0, 1]
Brake	[0, 1]
Steering	[−1, 1]

**Table 5:** Traffic density levels.

Density	Low	Medium	High	NO Traffic
Ratio	61%	33%	3%	3%

**Table 6:** Different speed limits in three different traffic densities.

	50 km/h	70 km/h	100 km/h
Low-density	21%	20%	20%
Mid-density	11%	11%	11%
High-density	1%	1%	1%
No-traffic	1%	1%	1%

**Table 7:** Hyperparameter set for hybrid autonomous traffic at un-signalized intersections.

Parameters	Value
The number of total training steps	1,000,000
The number of max steps per episode	200
Gamma	0.99
Clip parameter	0.2
Hidden layers	512
Batch size	2048
Learning rate	3 × 10^−5^
Optimizer	Adam

## Data Availability

The data that support the findings of this study are available from the corresponding author upon reasonable request.
